# Reconstruction for Limited-Projection Fluorescence Molecular Tomography Based on a Double-Mesh Strategy

**DOI:** 10.1155/2016/5682851

**Published:** 2016-10-17

**Authors:** Huangjian Yi, Xu Zhang, Jinye Peng, Fengjun Zhao, Xiaodong Wang, Yuqing Hou, Duofang Chen, Xiaowei He

**Affiliations:** ^1^School of Information Sciences and Technology, Northwest University, Xi'an, Shaanxi 710069, China; ^2^School of Life Science and Technology, Xidian University, Xi'an, Shaanxi 710071, China

## Abstract

Limited-projection fluorescence molecular tomography (FMT) has short data acquisition time that allows fast resolving of the three-dimensional visualization of fluorophore within small animal* in vivo*. However, limited-projection FMT reconstruction suffers from severe ill-posedness because only limited projections are used for reconstruction. To alleviate the ill-posedness, a feasible region extraction strategy based on a double mesh is presented for limited-projection FMT. First, an initial result is rapidly recovered using a coarse discretization mesh. Then, the reconstructed fluorophore area in the initial result is selected as a feasible region to guide the reconstruction using a fine discretization mesh. Simulation experiments on a digital mouse and small animal experiment* in vivo* are performed to validate the proposed strategy. It demonstrates that the presented strategy provides a good distribution of fluorophore with limited projections of fluorescence measurements. Hence, it is suitable for reconstruction of limited-projection FMT.

## 1. Introduction

Fluorescence molecular tomography (FMT) is a promising optical imaging tool to quantitatively determine the fluorophore distribution in animals [1, 2]. Because of its high sensitivity, low cost, and noninvasion, FMT has been successfully applied in cancer diagnosis, drug development, and therapeutics assessment [3–5]. FMT is accomplished by excitation of the fluorophore (such as fluorescent protein or fluorescent dyes) with lasers and collection of fluorescence light emitted from the fluorophore. The distribution of the fluorophore would be reconstructed from the fluorescent measurements collected by imaging system with an appropriate mathematical model [6–12].

The inverse problem of FMT involves reconstruction of the spatial fluorophore distribution inside the imaging domain from the collected data on the surface at the emission and excitation wavelengths. The reconstruction is severely ill posed due to the strong scattering of near-infrared photons propagation in biological tissues [13]. To alleviate the ill-posedness and get robust reconstructed images, great efforts have been made on reconstruction algorithms and imaging systems. Different regularization schemes have been proposed, for example, the frequently employed Tikhonov regularization and sparsity regularization, to improve the accuracy [14, 15]. Some* a priori* information, like anatomical information, optical properties, and permissible region, is incorporated in reconstruction. Anatomical information (provided by X-ray computed tomography, XCT [8], or by magnetic resonance imaging, MRI [16]) can be employed in the forward model of photon propagation or further inserted in the inversion problem in the form of priors to improve the regularization of the problem [17, 18]. A quantitative three-dimensional reconstruction of FMT can be implemented where the distribution of optical properties is obtained by diffusion optical tomography (DOT) [19]. Liu et al. present further studies on the effect of functional and structural* a priori* information on the accuracy of FMT [20]. Feasible region could reduce the scale of matrix equation of the inverse problem significantly, and it is also helpful for improving the quality of final results [21–28]. In [21, 22], the feasible region can be derived from the near-infrared measured boundary data. A region-shrinking strategy is utilized to make the feasible region gradually shrink from the whole imaging domain to a small region in [24]. In addition, feasible region can also be extracted from the previously computed procedure, and a mesh refinement scheme is further used in the feasible region [25–27]. This adaptive mesh scheme provides a good performance in reconstruction.

To obtain more measured data on the boundary, hybrid FMT/XCT imaging geometries collecting tomographic data over 360° projections have been reported and disseminated to researchers [8, 29]. However, collecting high spatial-sampling data at 360° projections usually require long time (roughly need 5 min~45 min, [1]), which is not suited for the visualization of fast biology processes* in vivo*. To address this problem, limited-projection-angle FMT or limited-projection FMT provides an effective way [30–32]. Limited-projection-angle FMT does not require rotating gantries but provides a subset of the information available to 360° system, which shorten the experimental time [30, 31]. Limited-projection FMT means the fluorophore is recovered using as few projections as possible, which accelerates data collection and reduces animal stress [32]. The challenge for limited-projection FMT is reducing the ill-posedness to obtain accurate and stable reconstructed images.

In this study, we developed a feasible region extraction method based on a double-mesh strategy for limited-projection FMT. Reconstruction for the inverse problem by *l*_1_-norm regularization is implemented with a coarse discretization mesh and a fine discretization mesh, separately. But the initial result with the coarse discretization mesh provides a rough region of fluorophore, which can be considered as a feasible region to guide the reconstruction on the fine mesh. Then the matrix scale on the fine mesh can be reduced largely. Furthermore, the final result on the fine mesh can be improved due to the feasible region.

The outline of this paper is summarized as follows. The photon propagation model in biological tissues and the proposed reconstruction strategy are introduced in Section 2. Numerical simulation experiments on a 3D digital mouse model and real small animal experiments are presented to validate our reconstruction strategy in Section 3. Discussion and conclusions are presented in Section 4.

## 2. Methods

### 2.1. Photon Propagation Model

Since the near-infrared photon propagation in biological tissues has the characteristics of high scattering and low absorption, the diffusion approximation to radiative transport equation (RTE) can well describe photon propagation through biological tissues [33]. In a continuous-wave (CW) form, the following coupled diffusion equations with Robin boundary condition are used to represent the photon propagation [33–35]:

(1) ∇·Dxr∇Φxr−μaxrΦxr=−Θδr−rs∇·Dmr∇Φmr−μamrΦmr=−Φxrημafrr∈Ω,

where subscript *x* and *m* denote excitation light and emission light, respectively. *D*(*r*) and *μ*_*a*_(*r*) denote the diffusion coefficient and absorption coefficient of tissues. Φ(*r*) is the photon density. The unknown fluorescence yield *ημ*_*af*_(*r*) is the parameter to be reconstructed, which is denoted as *X*(*r*) in the following part of this article. Using finite element method to solve (1) [36], for total *S* excitation point sources, we have the following final weighted matrix:

(2) $$\begin{array}{c} \Phi_{m}=AX, \end{array}$$

where *A* is *n* × *p* matrix, which establishes the linear relationship between the emitted fluorescence photon flux Φ_*m*_ ∈ *R* ^*n*^ on the surface and the unknown fluorescence yield distribution *X* ∈ *R* ^*p*^. The aim of FMT is to estimate *X* from the boundary measurements Φ_*m*_ with (2). More detailed descriptions can be found in [37].

### 2.2. Proposed Reconstruction Method

**Figure 1:**
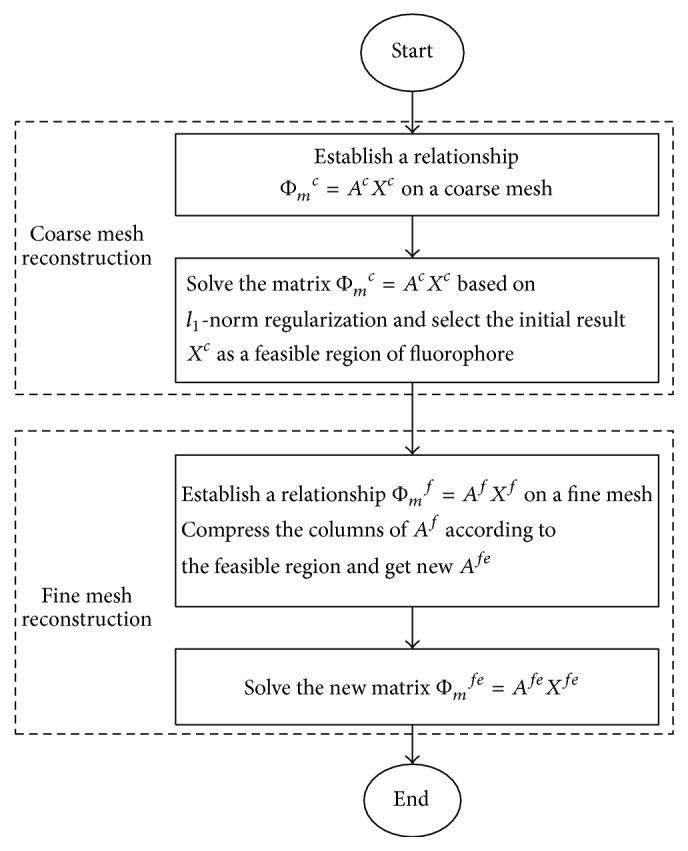
The flow chart of the proposed reconstruction method based on a double-mesh strategy.

Limited-projection FMT means the fluorophore is recovered using some projections, very few. So, the size of boundary measurements Φ_*m*_ is much smaller than the size of variable *X* (related to the nodes or tetrahedrons in the discretization mesh, typically the size around 10^3^~10^5^) in (2). It is a hard work to solve (2) directly; then this paper has developed a feasible region extraction strategy based on a double mesh for limited-projection FMT. Reconstruction for the inverse problem is implemented with a coarse discretization mesh and a fine discretization mesh, separately. First, a preliminary result is obtained rapidly on a coarse discretization mesh using *l*_1_-norm regularization. This initial result has low resolution due to the coarse discretization mesh, but it can be selected as a feasible region of fluorophore. To get a high resolution recovered image, a fine discretization mesh is utilized for the reconstruction problem although it results in enlarged variable *X*. To reduce the size of *X*, the feasible region has guided the reconstruction on the fine discretization mesh using *l*_1_-norm regularization. Here, *l*_1_-norm regularization is utilized based on the fact that the fluorophore is located in a certain area of interest in most FMT applications. It is in sparse pattern compared with the imaging domain [38]. The flow chart of the proposed reconstruction strategy is shown in Figure 1.

### 2.3. Quality Evaluation

To evaluate the quality of recovered images, center localization error (CLE), normalized root mean square error (nRMSE), relative error (RE), and contrast to noise ratio (CNR) are adopted in this study [39–41]. CLE is defined as

(3) $$\begin{array}{c} CLE={\left[\;{\left(\;x-x_{0}\;\right)}^{2}+{\left(\;y-y_{0}\;\right)}^{2}+{\left(\;z-z_{0}\;\right)}^{2}\;\right]}^{1/2}, \end{array}$$

where (*x*, *y*, *z*) is reconstructed center coordinate and (*x*_0_, *y*_0_, *z*_0_) is the actual center coordinate of fluorophore. nRMSE is defined as

(4) $$\begin{array}{c} nRMSE=\frac{\sqrt{{\left(\sum_{i=1}^{K}\left(X_{recon}\left(i\right)-X_{true}\left(i\right)\right)\right)}^{2}/K}}{\left(X_{recon}^{max}-X_{recon}^{min}\right)}, \end{array}$$

where *K* denotes the total number of the nodes. *X*_recon_(*i*) and *X*_true_(*i*) are the recovered values and the truth values on the *i*th nodes, respectively. *X*_recon_^max^ and *X*_recon_^min^ are the maximum and minimum recovered values. RE is defined as

(5) $$\begin{array}{c} RE=\frac{\left|X_{recon}-X_{true}\right|}{X_{true}}, \end{array}$$

where *X*_recon_ and *X*_true_ are the reconstructed and true fluorescence yield of fluorophore. CNR is defined as

(6) $$\begin{array}{c} CNR=\frac{\left(\mu_{ROI}-\mu_{ROB}\right)}{\sqrt{\left(\omega_{ROI}\sigma_{ROI}^{2}+\omega_{ROB}\sigma_{ROB}^{2}\right)}}, \end{array}$$

where *μ*_ROI_ is the mean value fluorescence yields in the region of interest (ROI) and *μ*_ROB_ is the mean value of fluorescence yield within the region of background (ROB). *ω*_ROI_ and *ω*_ROB_ are the number of the nodes in the ROI and ROB, respectively. *σ*_ROI_^2^ and *σ*_ROB_^2^ are the variances of fluorescence yields in the ROI and ROB. In general, a high-quality reconstructed image possesses CLE, nRMSE, and RE value close to 0 and a high CNR value.

## 3. Experiments and Results

In this section, numerical simulation experiments with a 3D digital mouse and real small animal experiments were designed to demonstrate the potential and feasibility of the proposed strategy for limited-projection FMT. We employed the incomplete variables truncated conjugate gradient method to solve (2), which has been demonstrated as an effective *l*_1_-norm regularization method in FMT [39].

### 3.1. Numerical Simulation Experiments

In this section, numerical simulation experiments were carried out on a 3D digital mouse model [42]. In general, the torso section of the mouse with a height of 35 mm was selected as the investigated region, which was composed of six organs: (1) muscle, (2) heart, (3) lungs, (4) liver, (5) stomach, and (6) kidneys. The specific optical properties are listed in Table 1 [36, 43].

Our reconstructions code written in MATLAB is conducted on a personal computer with a 3.40 GHz Intel® Xeon® CPU E3-1231 v3 and 8 GB RAM. A small sphere with a radius of 1 mm was to imitate the fluorophore, and it was located in the liver with the center coordinate (12.9 mm, 8.4 mm, 15.9 mm). The actual fluorescence yield of fluorophore was set to be 0.05 mm^−1^. For the forward problem, the torso model of digital mouse was discretized into 115,126 tetrahedral elements and 21,127 nodes to calculate the boundary measurements with the finite element method. The coarse mesh in the inverse has 2,993 nodes and 14,802 tetrahedral elements while the fine mesh has 8,101 nodes and 44,005 tetrahedral elements.

**Figure 2:**
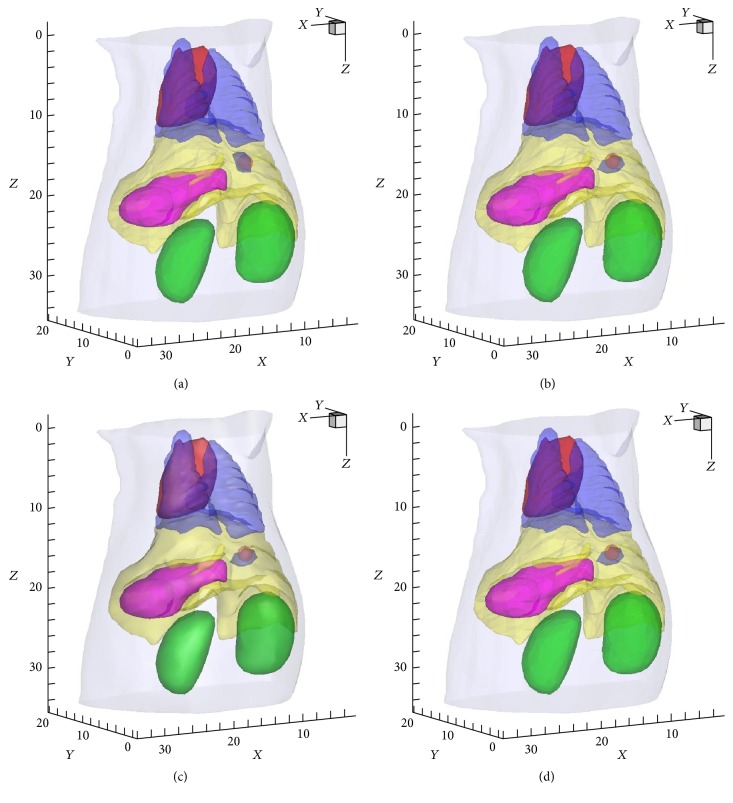
3D views of the reconstructed results with 3, 6, 9, and 12 projections, respectively.

**Figure 3:**
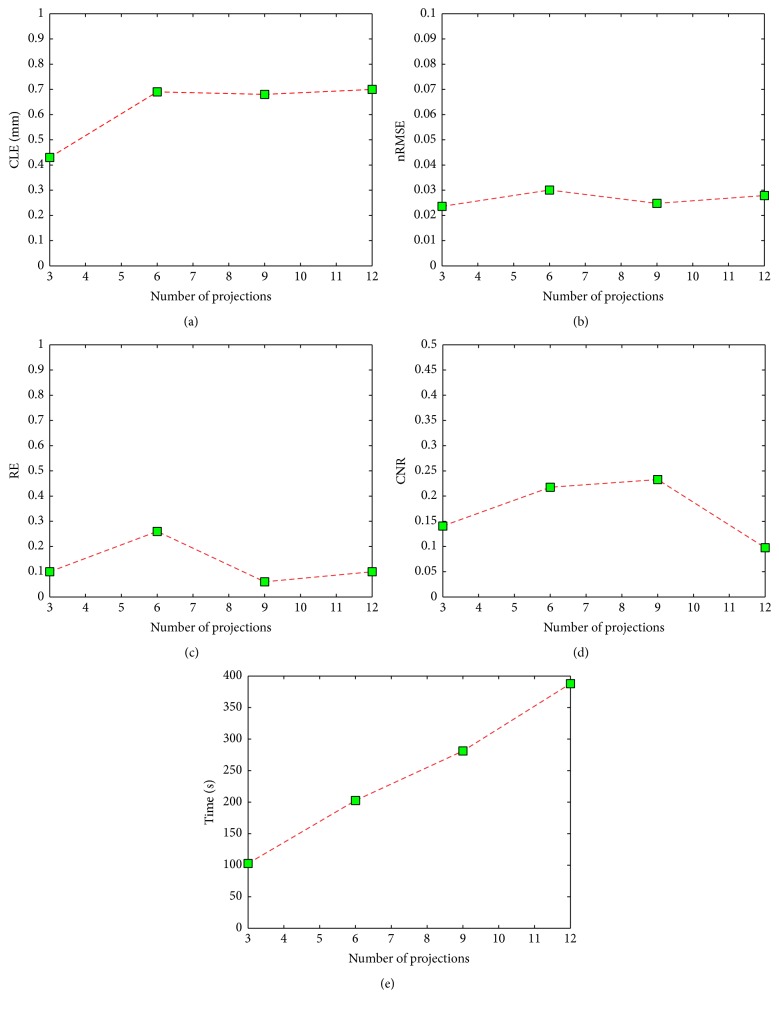
The quantitative results with 3, 6, 9, and 12 projections. (a) CLE (mm), (b) nRMSE, (c) RE, (d) CNR, and (e) time cost (s).

To demonstrate the possibility of the double-mesh strategy for limited-projection FMT, we investigated the FMT reconstruction with different projections. In fact, the influence of limited-projection on FMT has been studied comprehensively in [32]. Then a relationship between the recovered results with double-mesh strategy and projection numbers (3, 6, 9, and 12) has been presented. Excitation sources were positioned uniformly in a circle, and the field of view (FOV) of the detection with respect to each excitation source was 120° [39]. Here, the feasible region of fluorophore is determined from the initial results on the coarse mesh by choosing nodes with a threshold of 50% of the largest reconstructed fluorescence yield.  Figure 2 shows the 3D views of recovered results based on the double-mesh strategy with 3, 6, 9, and 12 projections. The corresponding quantitative results according to CLE, nRMSE, RE, CNR, and time cost (including the time spent in assembling the stiffness matrix and reconstruction) are presented in Table 2 and Figure 3. It is obvious that CLEs of 3, 6, 9, and 12 projections are smaller than 0.8 mm. There is no doubt the projection number of three costs the least time. From Table 2 and Figures 2(a) and 3(b)–3(d), the proposed strategy could provide acceptable values in nRMSE, RE, and CNR with 3 projections compared to other three cases. In [32], it is suggested that the projection number of 3 is preferred for fast FMT experiment. Then it indicates that the double-mesh strategy has the potential for limited-projection FMT.

**Figure 4:**
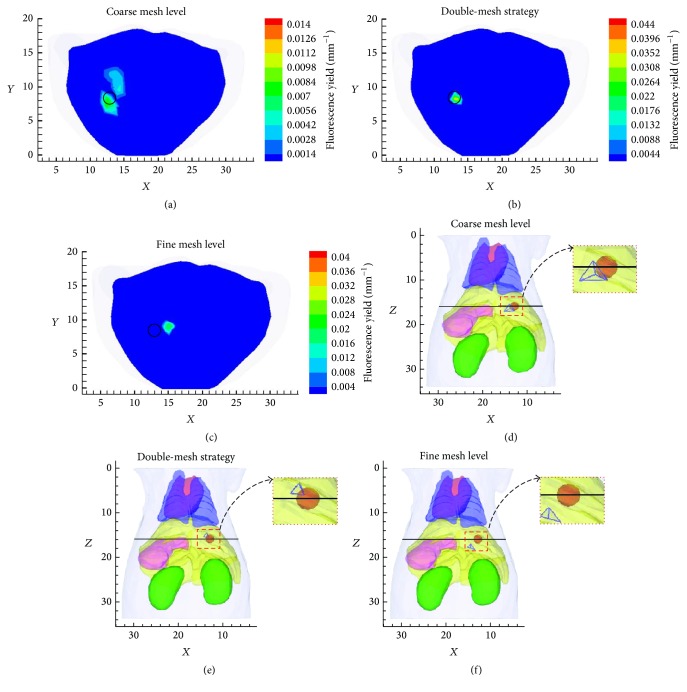
Reconstruction results with a coarse mesh, the double-mesh strategy, and the fine mesh based on *l*_1_-norm regularization. (a, b, c) show the cross-sectional views (*z* = 15.9 mm) of the fluorophore, and the black circles denote the real position of fluorophore. (d, e, f) show the corresponding coronal view of the recovered tetrahedral element, and the sphere is the fluorophore.

**Figure 5:**
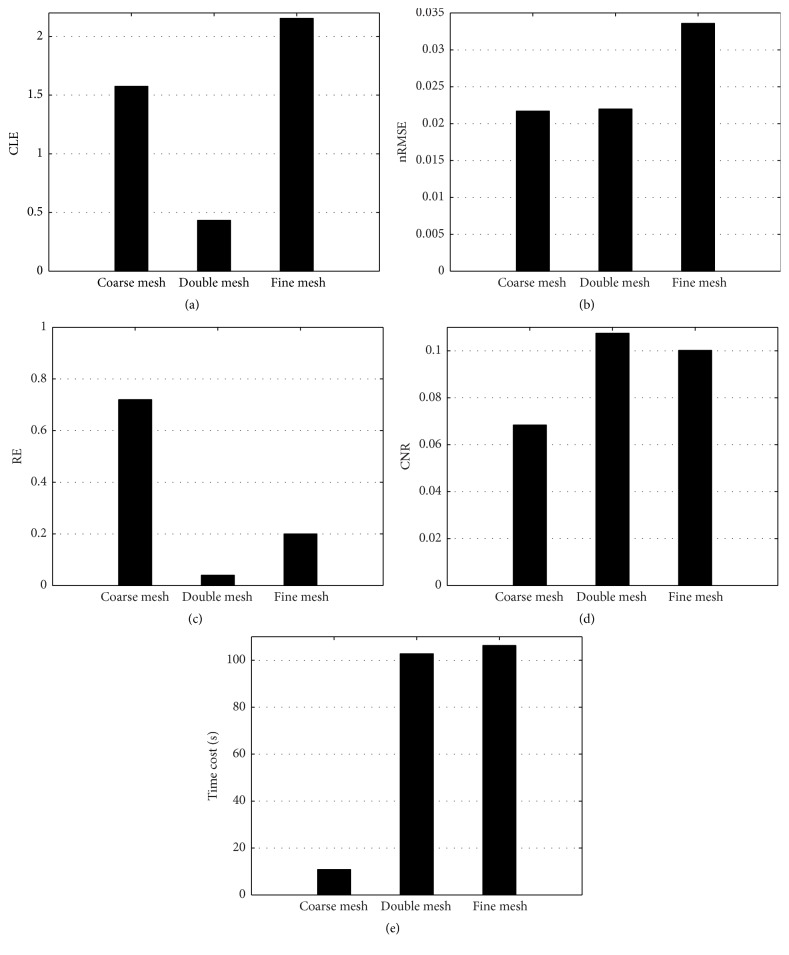
CLE (a), nRMSE (b), RE (c), CNR (d), and time cost (e) of recovered results for three mesh levels.

In order to further investigate the performance of the presented strategy, the reconstructed results with the double-mesh strategy (Figures 4(b) and 4(e)) are compared to the single mesh-based reconstruction (Figures 4(a) and 4(d) based on coarse mesh and Figures 4(c) and 4(f) based on fine mesh). Figures 4(a), 4(b), and 4(c) show the cross-sectional views (*z* = 15.9 mm), and Figures 4(d), 4(e), and 4(f) show the corresponding coronal view of the recovered tetrahedral element. It is obvious that the recovered image based on fine mesh is not accurate from Figures 4(c) and 4(f), even worse than the image in the coarse mesh. But the image quality is improved greatly after using the feasible region provided by the preliminary result based on coarse mesh, as shown in Figures 4(b) and 4(e).  Figure 5 shows recovered results according to CLE, nRMSE, RE, CNR, and time cost for three cases. The RE on the coarse mesh is large as shown in Figure 5(c), which means that the preliminary result is not accurate compared with the true fluorophore although it has the smallest computational time as shown in Figure 5(e). But the rough region is accurate enough to be selected as the feasible region to guide the fine mesh reconstruction. The fine discretization mesh can provide better spatial resolution of image, but fine mesh brought more variables in FMT which would aggravate the ill-posedness. This is the reason that the recovered image with fine discretization mesh has large values in CLE, nRMSE, and RE and small one in CNR. However, these parameters have been improved greatly after utilizing the feasible region which is provided by coarse mesh. It is the feasible region that improves the quality of results on the fine mesh. This is the key of the double-mesh strategy.

### 3.2. *In Vivo* Implanted Experiments

In this section, we further assess the performance of the developed strategy with* in vivo* small animal experimental data, which comes from [39]. A glass tube was implanted into the abdomen of an adult BALB/C to mimic the fluorescent target. It contains Cy5.5 solution (with the extinction coefficient of about 0.019 mm^−1^ *μ*M^−1^ and quantum efficiency of 0.23 at the peak excitation wavelength of 671 nm [44]) with 0.6 mm radius and 2.8 mm height. The true fluorescence yield of Cy5.5 is 0.0402 mm^−1^. The fluorescence data and anatomical information were collected by a noncontact continuous-wave FMT/micro-CT imaging system [39]. With micro-CT, the true center of the glass tube was (21.1 mm, 27.8 mm, 7.4 mm). The CT data were segmented into five major anatomical components, including heart, lungs, liver, kidneys, and muscle. The optical parameters for these five components at the excitation and emission wavelengths were calculated based on literature [43], shown in Table 3. Four excitation sources were positioned uniformly in a circle, which provided four projections of fluorescence measurements. The coarse mesh in the inverse has 3823 nodes and 18,504 tetrahedral elements while the fine mesh has 8,065 nodes and 43,481 tetrahedral elements.

**Figure 6:**
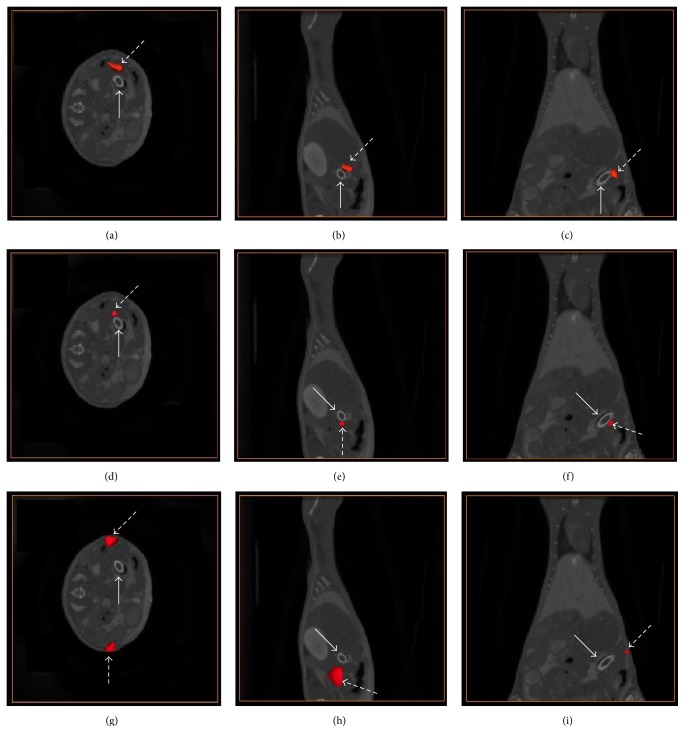
The reconstructed results with coarse mesh (a, b, c), double mesh (d, e, f), and fine mesh (g, h, i), respectively.

**Figure 7:**
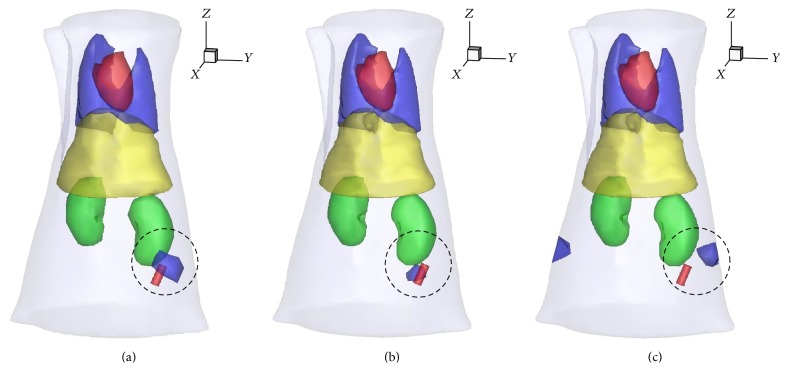
Recovered results in 3D views with coarse mesh (a), double mesh (b), and fine mesh (c). The red cylinder is the glass tube and the blue region is recovered target.

Figure 6 shows the recovered results overlaid with CT data. The red region (the dashed arrow) denotes the recovered tube, and the white ellipse is the true tube (the solid arrow). Figures 6(a), 6(d), and 6(g) are transversal slices, Figures 6(b), 6(e), and 6(h) are sagittal slices, and Figures 6(c), 6(f), and 6(i) are coronal views. Figures 6(a), 6(b), and 6(c) are reconstructed with coarse mesh by *l*_1_-norm regularization method, and its 3D view is shown in Figure 7(a). The recovered center of the tube is (21.4 mm, 29.1 mm, 8.5 mm) with CLE of 1.73 mm. Figures 6(d), 6(e), and 6(f) present results of the double-mesh strategy with 3D view in Figure 7(b). The recovered center of the tube is (20.4 mm, 28.6 mm, 7.0 mm), with CLE of 1.14 mm. Figures 6(g), 6(h), and 6(i) are reconstructed with fine mesh by *l*_1_-norm regularization method. It is obvious that there is a large error between the recovered and the true tube by the visual. It is consistent with 3D views in Figure 7(c). In fact, its CLE is 4.32 mm, which is much larger than that of the previous two.  Figure 7 is corresponding results for the three cases in 3D views, as shown inside the circle region. The red cylinder is the true target while the blue region is recovered target. The reconstructed result with fine mesh has the largest location error in the visual, which is consistent with Figures 6(g)–6(i). Furthermore, a spurious target also appeared, as shown in Figures 6(g) and 7(c), which may be caused by the ill-posedness of the problem. The reconstruction time for the coarse mesh, double mesh, and fine mesh is 39.71 s, 178.66 s, and 184.76 s.

## 4. Discussion and Conclusions

In this paper, we developed a feasible region extraction strategy based on a double mesh for limited-projection FMT. A preliminary result is rapidly obtained on a coarse discretization mesh, which is not accurate and has low resolution. But the rough region is accurate enough to provide a feasible region of fluorophore, which is very helpful to improve the reconstruction on a fine discretization mesh and reduce the computational cost of the reconstruction. First, we investigated the possibilities of reconstruction with limited-projection measurements. The relationship between image quality and projection number in the numerical experiments has shown that the proposed strategy can provide acceptable results according to CLE, nRMSE, RE, CNR, and time cost (including the time spent during assembling the stiffness matrix and reconstruction) with three projections. In addition, it is interesting that projection number of nine provides the smallest values in nRMSE and RE but biggest value in CNR compared to other three cases from Figure 3. It seems to show that projection number of nine is preferred for FMT with the double-mesh strategy. Second, the performance of the double-mesh strategy is compared to the reconstruction with single coarse mesh and fine mesh, respectively. It is noted that the image quality with fine discretization mesh is not good according to CLE, nRMSE, RE, and CNR. But these parameters have been improved greatly after utilizing the feasible region. Because *l*_1_-norm regularization provides a sparse result which includes only a few number of nodes with values, small nRMSE is obtained with coarse mesh. That is why coarse mesh and double mesh obtain similar nRMSE from Figure 5(b).* In vivo* small animal experiment has further demonstrated that the presented strategy has a potential in reconstruction of fluorophore with limited projections of fluorescence measurements.

This study has only focused on the single fluorescent target reconstruction model, while two or more targets' reconstruction model can be found in FMT applications. So our future work will focus on this research. In addition, the proposed method contains two reconstruction processes, namely, reconstruction on a coarse discretization mesh and on a fine discretization mesh. As we all know, the time for assembling stiffness matrix using finite element method is very large, which accounts for above 90% of the reconstruction time. To further reduce the computational cost, we will pay a great attention to the acceleration method on assembling the stiffness matrix of FEM. In conclusion, our strategy is suitable for limited-projection FMT.

## Figures and Tables

**Table 1:** Optical parameters of the mouse organs (units of *μ*_*a*_ and *μ*_*s*_′: mm^−1^).

Organs	*μ*_*ax*_	*μ*_*sx*_′	*μ*_*am*_	*μ*_*sm*_′
Muscle	0.0052	1.08	0.0068	1.03
Heart	0.0083	1.01	0.0104	0.99
Lungs	0.0133	1.97	0.0203	1.95
Liver	0.0329	0.70	0.0176	0.65
Kidneys	0.0660	2.25	0.0380	2.02
Stomach	0.0114	1.74	0.0070	1.36

**Table 2:** Quantitative results for 3, 6, 9, and 12 projections measurements.

Projections	CLE (mm)	nRMSE	RE	CNR	Time cost (s)
3	0.433	0.0236	10%	0.1406	102.77
6	0.687	0.0301	26%	0.2176	202.60
9	0.675	0.0248	6%	0.2328	281.09
12	0.695	0.0279	10%	0.0975	387.87

**Table 3:** Optical parameters of the mouse organs at 670 nm and 710 nm (units of *μ*_*a*_ and *μ*_*s*_′: mm^−1^).

Organs	*μ*_*ax*_	*μ*_*sx*_′	*μ*_*am*_	*μ*_*sm*_′
Muscle	0.075	0.412	0.043	0.350
Heart	0.051	0.944	0.030	0.870
Lungs	0.170	2.157	0.097	2.093
Liver	0.304	0.668	0.176	0.629
Kidneys	0.058	2.204	0.034	2.021
